# Editorial: Mapping microbial diversity onto the phylogeny of associated plant species

**DOI:** 10.3389/fpls.2024.1421637

**Published:** 2024-05-01

**Authors:** Qiu-Yun (Jenny) Xiang, Stephanie N. Kivlin, Douglas E. Soltis, Shixiao Yu, Haiyan Chu, Pamela S. Soltis, Yunpeng Zhao

**Affiliations:** ^1^Department of Plant and Microbial Biology, North Carolina State University, Raleigh, NC, United States; ^2^Department of Ecology and Evolutionary Biology, University of Tennessee, Knoxville, TN, United States; ^3^Department of Biology, University of Florida, Gainesville, FL, United States; ^4^Florida Museum of Natural History, University of Florida, Gainesville, FL, United States; ^5^Department of Ecology, School of Life Sciences, Sun Yat-sen University, Guangzhou, China; ^6^Institute of Soil Science, Chinese Academy of Sciences, Nanjing, China; ^7^College of Life Science, Zhejiang University, Hangzhou, China

**Keywords:** evolution of eastern Asian-North American disjunct plants, *Cornus*, Brassicaceae, tropical forest plant-microbial network, endophyte diversity, approaches impacting identification of mycorrhizae, plant-mycorrhizae fungal association specificity, role of plant phylogeny-genetics and habitat-distribution

Phylogeny provides the framework for studying functional traits within a clade of interest. Functional traits influence growth, survival, and reproduction by mediating interactions between the biotic and abiotic environments (Caruso et al., 2020). Linking evolution and ecology via a phylogenetic framework and the incorporation of functional traits represents a new goal of modern systematics. It is critical to understand the relationship of functional traits to the tempo and mode of evolutionary divergence of species as well as to the ecological processes of environmental adaptation and species coexistence (Ackerly, 2009). Our understanding of the diverse roles of functional traits in ecological processes remain limited. Understanding how functional traits shift in value during species diversification and how the pattern and rates of trait changes are affected by climate change, geographic isolation and phylogenetic constraints is critical to the study of lineage divergence and ecological adaptation. Microbial diversity associated with plants can be viewed as an ecological functional trait for study in systematics and evolution. Studies on this topic will shed light on the relationships and coevolution between plant diversity and associated microbial diversity. In this Research Topic, we assembled papers to stimulate more research on microbial diversity and plant functional traits in a phylogenetic context.

Endophytes can affect plant phenotype and whole ecosystem level processes (Christian et al. (2019); Harrison and Griffin, 2020. Knowledge of endophytes, their diversity and factors affecting their patterns and assembly in host plants, is fundamental to our understanding of plant-microbial interactions and how plant microbiomes affect stress tolerance. Assembling such a knowledge base is the 8th ranked major questions facing plant sciences (Amstrong et al., 2023), and is not only prerequisite toward understanding endophyte function in plants but will also guide studies regarding the mechanisms governing microbial community assembly. Despite numerous studies on endophytes, we know little about the effects of host plant phylogeny on endophyte diversity and evolution and conversely the role of divergence of endophyte diversity in host lineage diversification and speciation. In this Research Topic, Zhou et al. examined the patterns and possible drivers of allopatric divergence in sister species or clades (using *Cornus* as a model) isolated in Eastern North America and Eastern Asia, via study of foliar endophytes. They detected an overall significant, but relatively weak, correlation between endophyte community dissimilarity and phylogenetic distance of plants among the disjunct genera and between beta diversities of endophytes and phylogenetic distance of *Cornus* species. They also found large differences in endophyte communities among individuals within species, among genera, and among sampling locations. Their results suggested that host identity and environment are major factors in driving divergence of this functional trait, although phylogenetic divergence may also be important.

Within species, our knowledge about how endophyte diversity is affected by phylogeny or genetic differentiation, geographic isolation, and local environments of populations is also highly limited, although variation of endophyte diversity across sites or individuals has also been reported in some studies (Köberl et al., 2013; Wagner et al., 2016; Latz et al., 2021; Redondo et al., 2022; Su et al., 2023). In this Research Topic, Pais et al. made an innovative use of contaminant sequences from RAD-seq generated for range-wide genetic studies of flowering dogwood tree, *C. florida* L., from eastern North America to examine foliar fungal diversity and further assess the relationships of this diversity with genetics and environmental variables. They found that foliar fungal communities were shaped by both geographic-bioclimatic variation and genetic differentiation of host plants. The concepts and data processing workflow developed in the study may guide future research.

Mycorrhizal fungi acquire most host plants’ nutrients and water (Kakouridis et al., 2022). The selection and adaptation of mycorrhizal fungi and plant roots to each other are products of long-term ecological and evolutionary processes (Brundrett, 2002). However, our knowledge of the processes governing their assemblage in host plants is unknown in most plant lineages. The first step in creating a conceptual framework of plant-mycorrhizal fungal association specificity is adopting common definitions and tests of specificity among plants and their fungal consortia. In this Research Topic, d’Entremont and Kivlin review the evidence for plant host specificity for arbuscular and ectomycorrhizal fungi and propose a set of metrics to determine taxonomic, functional, and phylogenetic specificity of mycorrhizal fungal colonization among plant lineages. They proposed that the next step in predicting distributions of mycorrhizal fungal consortia is uncovering the environmental context dependency of plant-mycorrhizal fungal specificity. Li et al. explored the root-associated fungal pathogen community. Taking abiotic and biotic factors into account, they identified the roles of plant species identity, plant habitat, and plant mycorrhizal type in shaping the fungal pathogen community composition. Furthermore, they quantified the relative importance of different community assemblies and highlighted the determinant roles of functional traits. They found that plant species identity, plant habitat, and plant mycorrhizal type accounted for the variations in fungal pathogen community composition, with species identity and mycorrhizal type having dominant effects.

Zhu et al. investigated the effects of species abundance, spatial distribution, and phylogeny on a plant-ectomycorrhizal fungal network in a subtropical forest. They found that spatial overlap better predicted pairwise species interactions of plants and ectomycorrhizal fungi than species abundance. The strength of species interaction was significantly affected by the phylogeny of the ectomycorrhizal fungal species but not of plant species. They showed that all of these factors jointly drive the assembly of plant-ectomycorrhizal fungal networks. Studies on plant-microbial interactions are affected by available methodologies. Our ability to address long-term questions increases with the improvement of technologies for generating data and methods of data analyses. Our knowledge of plant-microbial interactions is continually renewed with new evidence from new studies. In this Research Topic, Trautwig et al. reviewed how different approaches have impacted the identification of mycorrhizae in Brassicaceae, and the ecological implication of mycorrhizae in the family. In contrast to previous studies, they discovered widespread colonization of AM fungi throughout Brassicaceae, suggesting that commonly held assumptions of Brassicaceae-produced fungicidal allelochemicals may have context-dependent effects on fungi across environments and Brassicaceae species.

## Author contributions

QX: Writing – review & editing, Writing – original draft, Supervision, Project administration, Investigation, Conceptualization. SK: Writing – review & editing, Investigation, Conceptualization. DS: Writing – review & editing, Supervision, Conceptualization. SY: Writing – review & editing, Conceptualization. HC: Writing – review & editing, Conceptualization. PS: Writing – review & editing, Supervision, Conceptualization. YZ: Writing – review & editing, Conceptualization.

## References

[B1] Ackerly D. (2009). Conservatism and diversification of plant functional traits: Evolutionary rates versus phylogenetic signal. Proc. Natl. Acad. Sci. U.S.A. 106, 19699–19706. doi: 10.1073/pnas.0901635106 19843698 PMC2780941

[B2] Amstrong E. M. Larson E. R. Harper H. Webb C. R. Dohleman F. Araya Y.. (2023). One hundred important questions facing plant science: an international perspective. New Phyto 238, 470–481. doi: 10.1111/nph.18771

[B3] Brundrett M. C. (2002). Coevolution of roots and mycorrhizas of land plants. New Phytol. 154, 275–304. doi: 10.1046/j.1469-8137.2002.00397.x 33873429

[B4] Caruso C. M. Mason C. M. Medeiros J. S. (2020). The evolution of functional traits in plants: is the giant still sleeping? Int. J. Plant Sci. 181, 1–8. doi: 10.1086/707141

[B5] Christian N. Herre E. A. Clay K. (2019). Foliar endophytic fungi alter patterns of nitrogen uptake and distribution in Theobroma cacao. New Phytol. 222, 1573–1583. doi: 10.1111/nph.15693 30664252

[B6] Harrison J. G. Griffin E. A. (2020). The diversity and distribution of endophytes across biomes, plant phylogeny and host tissues: how far have we come and where do we go from here? Environ. Microbiol. 22, 2107–2123. doi: 10.1111/1462-2920.14968 32115818 PMC7679042

[B7] Kakouridis A. Hagen J. A. Kan M. P. Mambelli S. Feldman L. J. Herman D. J.. (2022). Routes to roots: direct evidence of water transport by arbuscular mycorrhizal fungi to host plants. New Phytol. 236, 210–221. doi: 10.1111/nph.18281 35633108 PMC9543596

[B8] Köberl M. Schmidt R. Ramadan E. M. Bauer R. Berg G. (2013). The microbiome of medicinal plants: diversity and importance for plant growth, quality and health. Front. Microbiol. 4:400. doi: 10.3389/fmicb.2013.00400 24391634 PMC3868918

[B9] Latz M. A. C. Kerrn M. H. Sorensen H. Collinge D. B. Jensen B. Brown J. K. M.. (2021). Succession of the fungal endophytic microbiome of wheat is dependent on tissue-specific interactions between host genotype and environment. Sci. Total Environ. 759, 143804. doi: 10.1016/j.scitotenv.2020.143804 33340856

[B10] Redondo M. A. Oliva J. Elfstrand M. Bobergm J. Capador-Barreto H. D. Karlsson B.. (2022). Host genotype interacts with aerial spore communities and influences the needle mycobiome of Norway spruce. Environ. Microbiol. 24, 3640–3654. doi: 10.1111/1462-2920.15974 35315253 PMC9544151

[B11] Su J. Wang Y. Bai M. Peng T. Li H. Xu H.-J.. (2023). Soil conditions and the plant microbiome boost the accumulation of monoterpenes in the fruit of Citrus reticulata ‘Chachi’. Microbiome 11, 61. doi: 10.1186/s40168-023-01504-2 36973820 PMC10044787

[B12] Wagner M. R. Lundberg D. S. del Rio T. J. Tringe S. G. Dangl J. L. Mitchell-Olds T. (2016). Host genotype and age shape the leaf and root microbiomes of a wild perennial plant. Nat. Communication 7, 12151. doi: 10.1038/ncomms12151 PMC4945892 27402057

